# The Adverse Effect of Spasticity on 3-Month Poststroke Outcome Using a Population-Based Model

**DOI:** 10.1155/2014/696089

**Published:** 2014-07-24

**Authors:** S. R. Belagaje, C. Lindsell, C. J. Moomaw, K. Alwell, M. L. Flaherty, D. Woo, K. Dunning, P. Khatri, O. Adeoye, D. Kleindorfer, J. Broderick, B. Kissela

**Affiliations:** ^1^Departments of Neurology and Rehabilitation Medicine, Emory University School of Medicine, 80 Jesse Hill Jr. Drive SE, Atlanta, GA 30303, USA; ^2^Department of Emergency Medicine, University of Cincinnati, Cincinnati, OH 45219, USA; ^3^Department of Neurology and Rehabilitation Medicine, University of Cincinnati, Cincinnati, OH 45267, USA; ^4^Department of Rehabilitation Sciences, University of Cincinnati, Cincinnati, OH 45219, USA

## Abstract

Several devices and medications have been used to address poststroke spasticity. Yet, spasticity's impact on outcomes remains controversial. Using data from a cohort of 460 ischemic stroke patients, we previously published a validated multivariable regression model for predicting 3-month modified Rankin Score (mRS) as an indicator of functional outcome. Here, we tested whether including spasticity improved model fit and estimated the effect spasticity had on the outcome. Spasticity was defined by a positive response to the question “Did you have spasticity following your stroke?” on direct interview at 3 months from stroke onset. Patients who had expired by 90 days (*n* = 30) or did not have spasticity data available (*n* = 102) were excluded. Spasticity affected the 3-month functional status (*β* = 0.420, 95 CI = 0.194 to 0.645) after accounting for age, diabetes, leukoaraiosis, and retrospective NIHSS. Using spasticity as a covariable, the model's *R* ^2^ changed from 0.599 to 0.622. In our model, the presence of spasticity in the cohort was associated with a worsened 3-month mRS by an average of 0.4 after adjusting for known covariables. This significant adverse effect on functional outcomes adds predictive value beyond previously established factors.

## 1. Introduction

Spasticity is defined as a motor disorder characterized by a velocity-dependent increase in tonic stretch reflexes with exaggerated tendon jerks, resulting from hyperexcitability of the stretch reflex. The definition, however, fails to describe the complex etiologies of spasticity, especially after a stroke. It can be due to a loss of upper motor neuron function, changes in the properties of the muscle membranes, augmentation of prior reflexes, or a combination of the above.

Equally unclear is spasticity's effect on poststroke recovery. There are some reports which suggest adverse effects on recovery. In a longitudinal study of 95 subjects, Sommerfeld et al. found that, at 3-month poststroke, patients without spasticity had statistically significant better motor and activity scores than patients with spasticity [1]. However, there are others who argue that spasticity's effects are being overstated, specifically by mentioning the absence of evidence to suggest that treatment of spasticity improves stroke recovery [2]. In fact multiple medications and treatments of spasticity have been developed and are used routinely in the clinical setting for poststroke spasticity. The literature describes improvements in passive function only and reduction in disability after botulinum toxin administration to spastic muscles [3–5]. In their approval of botulinum toxin for poststroke spasticity, the FDA cited 3 studies which showed improvement in upper limb musculature tone as measured by Modified Ashworth Scale and physician global assessment of treatment response [6]. It is still unclear whether the treatments improve functional outcomes after a stroke.

Using data from the Greater Cincinnati/Northern KY cohort of stroke patients, we have previously published a validated multivariable regression model for predicting 3-month modified Rankin Scale (mRS) as an indicator of functional outcome [7]. In that model, variables such as baseline function, stroke severity, age, and periventricular white matter disease were found to be independent predictors of 3-month functional outcomes in stroke patients. Approximately 50% of outcome variance was accounted for by the model. We sought to determine the effect of spasticity on functional outcomes by adding this variable to our model, while describing the characteristics of those with and without spasticity after ischemic stroke in our cohort.

## 2. Methods

The Greater Cincinnati/Northern Kentucky Stroke Study (GCNKSS), a 5-county population-based study in Southwestern Ohio and Northern Kentucky, tracks the regional incidence of stroke and case fatality. This study was approved by the Institutional Review Boards at all participating institutions, and detailed methods have been previously described [8, 9].

As part of Phase III of the GCNKSS, a cohort of ischemic stroke patients was prospectively identified from the larger stroke population. After a potential subject was identified as having an ischemic stroke, the subject's treating physician was contacted for permission to approach the patient for informed consent. Informed consent was obtained either from the patient or from a proxy for patients who were unable to supply reliable information or were unresponsive, aphasic, or confused. The order of preference for a proxy was the spouse or live-in companion, adult child, parent, sibling, or close friend of the person. All ischemic stroke patients during 2005 at any of the 17 hospitals in our study area were eligible for enrollment; the primary reason for not enrolling was discharge before contact for consent.

For each case, trained research nurses abstracted demographics, presenting symptoms, functional status before stroke, social, family, and medical histories, medications (including treatment with t-PA as documented in the medical record), testing and laboratory results, and imaging studies. Data were recorded on case report forms. Stroke severity (retrospective NIH stroke scale score; NIHSS) was estimated from the medical record using the methods of Williams et al. [9], which we have subsequently validated [10].

Stroke team physicians reviewed each abstract and all available imaging studies to verify that each case was a stroke and to classify the subtype of stroke. This cohort was followed over time to determine short-term functional outcome which was assessed via an initial interview and a 3-month interview.

### 2.1. Initial Interview

Each consented patient or proxy underwent an initial face-to-face structured interview with a research nurse. The interview included questions about recent systemic illness, recent medications, past medical history, family history, and risk factors, including weight, eating behaviors, subjective stress ratings, and caffeine, alcohol, and tobacco use.

### 2.2. Three-Month Interview

At 3 months after stroke, research nurses telephoned patients or proxies and asked about vital status, poststroke hospitalizations, medical contacts other than simple office visits, and current residence. The modified Rankin Scale (mRS) was used to determine the functional status of each surviving patient.

### 2.3. Assessment of Spasticity

Assessment of spasticity occurred at the 3-month direct interview. Spasticity was defined by a positive response to the question “Did you have spasticity following your stroke?” Prior to soliciting responses the interviewers described spasticity using its commonly occurring signs and symptoms if necessary. Medications for controlling spasticity were also documented.

### 2.4. Mortality

Mortality was assessed by use of Ohio and Kentucky death records (complete through 2003). The Social Security Death Index was searched via RootsWeb for deaths not already found in the Ohio and Kentucky records. Deaths found via chart review were verified by one or more of the three aforementioned sources.

### 2.5. Statistical Analysis

Linear regression was used to predict the probability of death and logistic regression was used to predict functional outcome at 3 months. The mRS was our primary measure of functional status and is presented below. For modeling 3-month mortality and functional outcome, univariable analyses identified independent predictors from among clinically relevant variables that were reasonably available to physicians during the acute hospitalization after stroke admission, that is, demographics, medical history, acute imaging results, acute treatments, stroke severity scores (retrospective NIHSS), and measures of functional independence (mRS). Only these variables were considered in building the primary model for predicting 3-month outcomes. The effects of comorbidities that occurred during the 3-month period were considered separately, as modifiers of the predicted outcome. Although poststroke therapy (physical, occupational, and speech therapy) might also modify outcome, it was not included in the model because of its bidirectional effect; patients with excellent or very poor poststroke status were both unlikely to receive therapy.

Significant predictors were then combined into a single model, and nonsignificant terms were removed using a manual backwards stepwise procedure [7]. For all modeling, colinearity of predictor variables was evaluated to minimize the likelihood of inappropriate inferences. At each stage of model development, the primary criterion for removal was a significance level less than 0.05. The impact of a variable's removal was gauged by inspection of the regression parameter estimates to ensure that interactions and spurious relationships were not evident. In addition, because removing a variable based solely on significance level might result in a large change in model accuracy, nonsignificant variables were not removed if the* C*-statistic (for logistic regression) or the *R* ^2^ (for linear regression) changed more than 0.1. For this analysis, we added spasticity to our model and tested whether its inclusion improved model fit. Analyses were conducted using SPSS version 14.0 (SPSS Inc.).

The functional outcome we modeled was the mRS, a 6-level ordinal variable. We elected to use linear regression for simplicity of interpreting our results. The rationale for using this statistical model and the mRS as the outcome measure has been explained previously [7]. We allowed the parameter estimates to vary but initially maintained the same variable structure. We then substituted spasticity as dichotomous variable (presence versus absence) and reexamined model performance. Patients who had expired by 90 days (*n* = 30) or did not have spasticity data available (*n* = 102) were excluded from the analysis.

## 3. Results

460 ischemic stroke survivors were interviewed in hospital and at 3 months. The mean age of the cohort was 67 years (SD = 14). 52% were male and 25% were black. The median NIHSS was 4 with an IQR = (2,7). Seventy-three subjects (22%) reported having a prior stroke. At 3-month poststroke, 30 subjects had died and 102 were either lost to follow up or did not answer the spasticity question. Thus, 328 remaining subjects were included in this analysis. Fifty-four of the 328 reported spasticity (16.5%, 95 CI 12.7–21.0). Twelve subjects (3.6%) were treated for their spasticity with 11 of these being treated with medications.

Table 1 shows the differences between subjects with spasticity and those without it in the cohort. The subjects with spasticity were younger and had slightly more severe strokes as measured by the NIHSS. There were not any differences between the two groups regarding potential confounding variables such as gender and diabetes; prior strokes and severe periventricular white matter disease were statistically insignificant. Both groups had similar baseline and immediate poststroke mRS scores. However, the subjects with spasticity had a higher 3-month mRS compared to those without it indicating a higher level of disability.

The results of our modeling are shown in Table 2. Among the 328 patients for whom spasticity was assessed at 3 months, the occurrence of spasticity additionally affected the three-month functional status (*β* = 0.420, 95 CI 0.194 to 0.645). This suggests that patients with spasticity have a higher modified Rankin Scale by 0.42 (just under half a point on average) at 3 months following stroke as compared to patients without spasticity after adjusting for confounders. With spasticity included as a variable, the model *R* ^2^ increased from 0.599 to 0.622.

## 4. Discussion

We used a prospectively enrolled cohort of ischemic stroke subjects drawn from a population-based epidemiology study to investigate the occurrence of characteristics of stroke patients with spasticity and the effect of spasticity on poststroke clinical function as measured by the mRS. Our results demonstrate that approximately 16% of subjects in our cohort had an occurrence of spasticity 3-month poststroke. This 3-month proportion is comparable to previously reported number of 19% by Sommerfeld et al. [1]. Other studies have documented higher percentages but the spasticity occurrence was measured at longer time intervals poststroke [11–13]. Approximately 22% the subjects with spasticity received treatment for their spasticity, representing 4% of the entire cohort. The subjects with spasticity were younger and had more severe strokes. This age factor has not been reported previously but the severity of stroke, particularly severity of paresis, has previously been reported as a factor [14].

Our data suggest that there is a significant difference in functional outcome as measured by the mRS at 3 months, where spasticity is associated with worse outcomes. There are several hypotheses to explain and support this finding. One can logically deduce that spasticity adversely affects motor function, which in turn can affect overall function. Support comes from the Sommerfeld study [1] where subjects without spasticity had statistically significant better motor and activity scores than those with spasticity. Specifically, correlations between increased muscle tone and the motor and activity scores were found for the initial upper-extremity Modified Ashworth Scale (MAS) and Birgitta Lindmark Motor Assessment (BL) active movement scores, the 3-month upper-extremity MAS and BL active movement scores, and Nine-Hole Peg test scores. However, the correlation was insignificant for other measures in the study. In another study of the same cohort [15], upper-extremity spasticity was associated with worse BL scores but lower- extremity spasticity was not associated with it. Differences in results between the above- mentioned studies and our study are likely due to the small numbers in the Sommerfeld cohort as well as choice of outcome measures. Whereas the modified Rankin scores are a measure of overall function, the BL and Nine-Hole Peg tests are specific motor outcome measures.

Our results of spasticity on 3-month outcomes are consistent with results from studies at a longer poststroke duration. Watkins et al. demonstrated that patients with spasticity were more likely to receive institutional care and had significantly lower BI scores at 12 months (*P* < 0.0001) [11]. Brashear et al. found predominantly moderate- to-high correlations between the MAS scores and the functioning scores (BL, RMI, and BI) at 18-month follow-up [5]. Lundström et al. assessed impaired function using the National Institutes of Health Stroke Scale (NIHSS) [16]. Disability related to activity performance and participation was assessed using the modified Rankin Scale (mRS) and BI. NIHSS scores were higher in patients with spasticity as compared to patients without spasticity one year after stroke. All patients with spasticity exhibited some degree of paresis. The proportion of patients with dependence in everyday activities according to mRS scores and BI was greater for patients with spasticity than for patients with no spasticity.

Given the shorter time interval poststroke of our study, the data has clinical relevance. It shows that spasticity can be identified in patients and, given its effect on outcome, it should be addressed. Since many of the patients during the 3-month interval poststroke would still be in active poststroke therapy compared to those 12–18 months out poststroke, we would argue that spasticity could and should be readily addressed using current therapy techniques as well as medications to improve function. Given that only a small number of subjects in our study reported use of medications to address spasticity, their effect on improving outcome could not be addressed.

One limitation of this study is that spasticity was assessed by asking the patients for their subjective opinion of whether they had spasticity. Although this brings some limitations, this patient-based methodology may also have some merit. There are not any generally accepted tools available to assess spasticity by telephone or without requiring a physical exam. Our questioning is subject to recall bias as well as information bias. In other studies, examining spasticity using epidemiological methods, spasticity is quantified using scales such as the Modified Ashworth Scale or the Tonal Assessment Scale. However, we are reassured by the fact that the prevalence of spasticity measured in our cohort is comparable to other studies. Further research as to the best way to assess spasticity without examination is needed and would be of great utility for future studies.

## 5. Conclusion

Our data adds to the small but growing literature on the prevalence of poststroke spasticity and its effect on outcomes. Specifically, this is the first report of the occurrence of spasticity in a United States based cohort and the occurrence of spasticity at that 3-month time period is comparable to previous literature at the same time period. Furthermore, we report that spasticity was associated with worse 3-month outcomes and is an independent predictor of worse outcome in our previously published theoretical model.

## Figures and Tables

**Table 1:** Characteristics of patients with and without spasticity.

	No spasticity		Spasticity		*P* value
Age at time of stroke	68	25–93	59	29–89	<0.001

Black	63	23.0	21	38.9	0.100
Nonblack	211	77.0	33	61.1	

Female	134	48.9	25	46.3	0.767
Male	140	51.1	29	53.7	

Diabetes	103	37.6	22	40.7	0.759
Prior stroke	56	20.4	17	31.5	0.106
Severe periventricular white matter disease	50	18.5	5	9.4	0.159

Estimated NIHSS	4.0	0–23	6.0	0–25	0.019

Prestroke Rankin	1.0	0–4	1.0	0–4	0.667
Poststroke Rankin	3.0	0–5	3.0	1–5	0.003
3-month Rankin	2.0	0–5	3.0	1–5	0.000

Numbers are median and range or frequency and percent.

**Table 2:** Multivariable logistic model predicting mRS at 3 months.

Parameter	*β*	95% CI interval (*β*)		*P* value
Model not including spasticity, *R* ^2^ = 0.599				
Diabetes	0.01	−0.15	0.17	0.901
Severe PVWMD	−0.14	−0.36	0.08	0.203
Age (per year)	0.00	0.00	0.01	0.683
Prestroke Rankin	0.28	0.20	0.36	0.000
Poststroke Rankin	0.59	0.51	0.67	0.000
Estimated NIHSS	0.02	0.00	0.04	0.015

Model including spasticity, *R* ^2^ = 0.622				
Spasticity	−0.42	−0.65	−0.19	0.000
Diabetes	−0.03	−0.20	0.14	0.758
Severe PVWMD	−0.21	−0.44	0.01	0.061
Age (per year)	0.00	0.00	0.01	0.229
Prestroke Rankin	0.25	0.17	0.33	0.000
Poststroke Rankin	0.58	0.50	0.67	0.000
Estimated NIHSS	0.02	0.00	0.04	0.023
